# Identification of Blau Syndrome disease signatures

**DOI:** 10.1186/1546-0096-9-S1-O25

**Published:** 2011-09-14

**Authors:** KP Foley, L Wang, D Cooper, M Magid-Slav, D Lipshutz, J Connor, M Miller, B Votta, P Gough, X Valencia, CH Wouters, CD Rosé, J Bertin

**Affiliations:** 1 Pattern Recognition Receptor Discovery Performance Unit, GlaxoSmithKline, Collegeville, Pennsylvania, USA; 2 University of Leuven, Leuven, Belgium; 3 Alfred I. duPont Hospital for Children, Wilmington, Delaware, USA

## Background

Blau Syndrome is an inherited granulomatous inflammatory disorder caused by gain-of-function mutations in NOD2, leading to activation of downstream signaling through the RIP2 kinase and production of pro-inflammatory cytokines.

## Aim

To identify robust biomarkers of disease activity, we undertook a systematic analysis of whole blood gene and plasma protein expression in Blau patients versus normal healthy volunteers (NHVs).

## Methods

Whole blood cell total RNA and plasma samples were obtained from Blau patients (*N*=6) and NHVs (*N*=8) and used in accord with informed consents. Transcriptional profiling was performed using Affymetrix microarrays, and immunoassays for 261 plasma proteins were performed using multiplexed Luminex arrays. Results were confirmed by real-time RT-PCR and ELISAs on independent samples.

## Results

By transcriptional profiling, a 21-gene signature was identified as differentially expressed between Blau and NHV samples. Consistent with constitutive activation of NOD2 signaling in Blau, 14 out of 21 of these genes were similarly regulated by NOD2 ligand stimulation in a NHV whole blood assay (*P*<0.0001). Plasma protein profiling identified a 7-protein signature specifically upregulated in Blau relative to NHV samples. Cross validation by PLS-DA supervised classification showed 90-100% accuracy in assigning patient disease status. Of particular interest, the inflammatory protein S100A12 (EN-RAGE) was found to be increased by 4.6 fold in Blau plasma (FDR *P*=0.005).

## Conclusion

We have identified both whole blood transcriptional and plasma protein signatures that uniquely distinguish Blau from normal volunteers. S100A12, a recently emerging biomarker of neutrophil-mediated inflammation, is consistently upregulated in Blau patients and may be a useful biomarker for monitoring disease state in future clinical trials. Finally, bioinformatic pathway analyses and comparisons of these findings with other known disease signatures may provide new insights into the pathogenesis of Blau Syndrome.

